# Prognostic significance of clinicopathological parameters, margin width and locoregional recurrences on outcome of primary and radiation associated breast angiosarcoma- results from a large UK sarcoma regional service

**DOI:** 10.1186/s13058-026-02265-0

**Published:** 2026-03-28

**Authors:** Samar Ali, Salena Bains, Emily Fox, Anant Desai, Mike Hallissey, Alaa El-Ghobashy, Robert Warner, Abeer M. Shaaban

**Affiliations:** 1 Cellular Pathology, Queen Elizabeth Hospital Birmingham, Birmingham, UK; 2 Oncoplastic Breast Surgery, Queen Elizabeth Hospital Birmingham, Birmingham, UK; 3 Obstetrics and Gynaecology, Birmingham Women’s Hospital, Birmingham Women’s and Children’s NHS Foundation Trust, Birmingham, UK; 4 Midlands Abdominal and Retroperitoneal Sarcoma Unit, Queen Elizabeth Hospital Birmingham, Birmingham, UK; 5 Department of Gynaecological Oncology, Obstetrics and Gynaecology, The Royal Wolverhampton Hospitals NHS Trust, Wolverhampton, UK; 6 Plastic and Reconstructive Surgery, Queen Elizabeth Hospital Birmingham, Birmingham, UK; 7 Department of Cancer and Genomic Sciences, University of Birmingham, Birmingham, UK

**Keywords:** Breast angiosarcoma, Radiation associated angiosarcoma, c-Myc

## Abstract

**Background:**

Breast angiosarcomas (AS) are rare and aggressive malignancies, categorised as primary angiosarcoma (PAS) and radiation-associated angiosarcoma (RAAS). Due to its rarity, large series of PAS and RAAS are limited. We aimed to analyse the outcome of a large cohort of angiosarcomas with emphasis on the prognostic factors, latency, margin width and significance of locoregional recurrences.

**Methods:**

A retrospective study was conducted on angiosarcomas managed at a large UK Regional Sarcoma Centre between 2013 and 2024. Clinical, pathological, disease-free survival (DFS) and overall survival (OS) data were collected. The interval between radiotherapy delivery and the development of angiosarcoma was calculated. Cox regression models and binary logistic regression were utilised for optimal threshold values for resection margins and patient outcomes.

**Results:**

PAS presented at a younger age (median 31 vs 71 years for RAAS). A significant shortening of latency between radiotherapy and onset of RAAS was found (r = − 0.719, *p* < 0.001). DFS and OS were 59.6%, and 54.2% respectively. Smaller microscopic resection margins were significantly associated with recurrence (*p* = 0.001). Recurrence was a strong predictor of mortality, HR = 2.856, p = 0.005. Mean survival with and without angiosarcoma recurrences was 32.9 and 80.5 months respectively (*p* = 0.005). Resection margins ≥ 10 mm were significantly associated with lower rates of recurrences (*p* = 0.044, OR = 0.323). However, resection margin distance did not directly impact survival (HR = 1.002, *p* = 0. 798). Cox regression analysis showed angiosarcoma size was not a predictor of survival (*p* = 0.278, HR = 1.002). Neither patient age nor the angiosarcoma histological grade correlated with recurrences or patient survival. C-Myc immunohistochemistry was positive in four of nine (44.44%) PASs and in 95.52% of RAAS. Its expression did not correlate with patient survival.

**Conclusion:**

Over the last decade, the number of diagnosed RAAS cases has increased with a shortening in the interval between radiotherapy and onset of RAAS. Margin status and recurrences, but not angiosarcoma grade or size, impact survival. Achieving a clear surgical margin is therefore critical for improved patients’ outcome.

## Introduction

Angiosarcomas (AS) are a rare, aggressive endothelial cell malignancy arising from lymphatic or vascular origins [1, 2]. They account for approximately 1% of all soft tissue sarcomas [2, 3]. AS of the breast is categorised into either primary (PAS) or radiation associated angiosarcomas (RAAS). PAS of the breast describes lesions which arise de novo, with no history of breast radiotherapy [4, 5]. These account for around 20% of all breast AS [5] and 0.05% of all breast malignancies [6]. RAAS of the breast are a rare but significant and well recognised long-term sequelae of adjuvant radiotherapy following surgical management of breast cancer [3, 6]. Those who develop RAAS are usually older, post-menopausal women (median age of 70), compared to those with PAS [7].

The clinical presentation of breast AS is variable. However, PAS often presents as subcutaneous masses, while patients with RAAS are more likely to present with cutaneous lesions [8]. Due to the variability in the clinical presentations and the rarity of the disease, diagnosis may be delayed. Immunohistochemistry using c-Myc positivity is thought to be useful in distinguishing PAS and RAAS [4, 8], however, newer research has challenged this view [9].

The British Sarcoma Group (BSG) guidelines [10, 11] recommend that all cases be discussed within a sarcoma MDT (multidisciplinary team) and surgical resection should be performed by an appropriately trained surgeon. Excision margins of greater than 10 mm are considered acceptable as a consensus [12], but the macroscopic margin required to achieve a microscopically clear margin and the impact of margin width on RAAS recurrence have remained controversial [13, 14]. Unfortunately, despite complete resection, local recurrence rates are high with a high likelihood of metastasis. Delayed diagnoses, challenges in management and the aggressive nature of the disease result in poor outcomes [15]. The 5-year survival rate is around 10% [12]. However, recent studies have shown the involvement of sarcoma MDT [16] and management in specialist centres [12] had superior outcomes compared to those managed in local centres increasing the 5-year survival to up to 50%.

Several studies, of generally small numbers, have provided conflicting conclusions on the histological factors associated with patient survival [4, 17]. There is currently limited literature and evidence base for the management of breast AS. While surgery remains the cornerstone, other modalities including neoadjuvant and adjuvant chemotherapy have been proposed, though with limited impact on survival [14]. In addition, there is no consensus on the optimal margin width that achieves R0. Despite different treatment modalities, local recurrence rates ranged from 40 to 90% leading to poor outcomes [18].

The aim of this study, therefore, is to assess clinical and pathological factors, including microscopic margin width, that may affect prognosis of breast AS, and investigate the latency period between radiotherapy delivery and RAAS development. This was done through analysis of a large well-characterised cohort of lesions managed at a UK tertiary referral and regional sarcoma service.

## Methods

An audit was approved and registered at the institution’s Clinical Audit Registration & Management System. All patients referred to a regional UK tertiary centre (Midlands Breast & Sarcoma Service) diagnosed with RAAS of the breast between February 2013 and March 2024, with preceding breast cancer regardless of its date, were identified from the breast oncoplastic surgical database. Patients referred to the Service but subsequently found to have other conditions, such as atypical vascular lesions or other radiation associated sarcomas, were excluded from this study.

The institutional patient electronic records and pathology database were used to collect the following data: clinical presentation of the angiosarcoma, surgical management, histological features including c-Myc expression, recurrences, survival duration and date of death if applicable. Detailed information on the preceding breast cancer and interval between breast cancer and angiosarcomas diagnoses were collected. Where the primary breast cancer was diagnosed and treated elsewhere, data was requested and collected from the referring hospital.

The diagnoses were confirmed histologically by central pathology review if the biopsy originated elsewhere. The therapeutic resections were all performed at the regional service and specimens handled and reported by specialist pathologists as per local protocols. All cases were discussed at both the sarcoma and breast multidisciplinary team meetings.

### Statistical analysis

Data on latency of RAAS development, tumour size, resection margin and recurrence were analysed. Where the exact month of diagnosis was unknown, June was used as a midpoint for consistency. The latency of RAAS development was calculated from the year the patient had received adjuvant radiotherapy for their primary breast cancer, to the year they first developed RAAS. Where more than one episode of breast cancer occurred, the date of the latest ipsilateral episode associated with radiotherapy administration was used.

Survival data was collected in months from initial angiosarcoma diagnosis until death or date last seen. For deceased patients, the cause of death (angiosarcoma related or otherwise) was obtained from the electronic patient record and General Practitioner (GP) databases. Data was censored to the date patient last seen for any medical reason.

The greatest microscopic dimension of the lesion was used for tumour size and the nearest microscopic resection margin was used for resection margin analyses. Logistic regression analysis and Cox regression models were performed to assess the impact of the microscopic surgical resection margin width on the risk of recurrence (binary outcome: recurrence vs. no recurrence).

Spearman’s rank order correlation test was used to identify significant correlation between variables including the correlation between year of radiotherapy and the diagnosis of angiosarcoma. The 5 year overall and disease specific survival were calculated using the Kaplan-Meir method. P values of less than or equal to 0.05 were considered statistically significant. IBM SPSS statistics software version 31 was used for statistical analyses.

## Results

A total of 79 female patients were included in this study, of whom 70 were diagnosed with radiation induced angiosarcoma (RAAS), including 4 patients with bilateral RAAS. Nine patients were diagnosed with PAS, 8 unilateral and one case of bilateral PAS.

Table 1 summarises the presentation and clinicopathological parameters of the study patients.

**Table 1:** Baseline clinicopathological features and cox regression analysis of RAAS cohort

Parameter	
Number of patients	70
Unilateral RAAS	66 (94.3%)
Bilateral RAAS	6 (8.57%)
Age at diagnosis: median (range)	71 (55–93)
Latency median (range)	8 (3–24)
*Tumour characteristics*	
Tumour size, mm; median (range)	65 (10–270)
Nearest resection margin, mm; median (range)	15 (0–85)
Local and/or distant recurrence	22 (31.4%)
*c-Myc expression*	
Strongly positive (of 67 tested)	64 (95.52%)
Weakly positive	3 (4.48%)
Not tested	3 cases
Ki-67 ≥ 70%	46 (65.7%)
Axillary lymph node involvement	2 cases (both bilateral RAAS)
*Treatment of RAAS*	
Surgery alone (extended mastectomy)	55 (78.6%)
Surgery + adj chemotherapy	5 (7.1%)
Surgery + adj radiotherapy	3 (4.0%)
Neoadjuvant chemotherapy alone	1 (1.4%)
Palliative treatment only	6 (8.6%)
*Outcomes*	
Deaths (all causes)	38 (54.3%)
5-year disease-specific survival	59.6%
5-year overall survival	54.2%
Median interval from RAAS diagnosis to death in months (range)	26 (3–180)
Mean survival with recurrence	32.9 months
Mean survival without recurrence	80.5 months
*Cox Regression Analysis (survival: dead vs alive)*	
Univariate: Margin width → recurrence	OR = 0.935, *p* = 0.006
Univariate: Margin ≥ 10 mm	OR = 0.323, *p* = 0.044
Univariate: Margin width → survival	HR = 1.002, *p* = 0.798 (NS)
Univariate: Tumour size → survival	HR = 1.002, *p* = 0.278 NS
Univariate: Recurrence → mortality	HR = 2.856, *p* = 0.005
Multivariate: Margin width	HR = 1.002, *p* = 0.798 (NS)
Multivariate: Tumour size	HR = 1.002, *p* = 0.278 (NS)
Multivariate: Recurrence	HR = 2.856, *p* = 0.005
Multivariate: Grade	NS
Multivariate: c-Myc	NS

*RAAS* Radiation associated angiosarcoma, *NS* non-significant

### Radiation associated angiosarcomas (RAAS) presentation

Of the 70 patients with a diagnosis of RAAS, the median age at diagnosis was 71 years (range 55–93 years). Where stated, the commonest presentations were masses, lumps or nodules, which were either fungating, haemorrhagic or purple in colour. Those presenting with skin changes most commonly presented with breast skin discolouration.

### Histological features and c-Myc expression

Histological appearances ranged from well differentiated vasoformative lesions to poorly differentiated epithelioid or spindle cell angiosarcoma. 64 cases (95.52%) tested strongly positive for c-Myc immunohistochemistry, 3 (4.48%) were weakly positive and data was not available on 3 patients. 65.7% of RAAS cases had a Ki-67% of 70% and above with some tumours exhibiting a 100% proliferation index. Axillary lymph node involvement was identified in two cases; both of which were of bilateral RAAS.

### Bilateral RAAS angiosarcoma

A total of 4 cases presented with bilateral RAAS, 2 were metachronous and 2 synchronous disease. The preceding breast cancer was bilateral in all 4 patients with all episodes of breast cancer treated by surgery followed by radiotherapy. Only one patient remains alive, 6 years following surgery. Table 2 summarises the presentation and outcome of patients with bilateral RAAS.

**Table 2:** Summary of presentation and outcome of bilateral radiation associated angiosarcoma

Patient	Breast cancer	RAAS Metachronous/ Synchronous	Latency (yrs)	Clinical details	RAAS surgery	Outcome
1	Bilateral synchronous breast cancer	Synchronous	9	Breast skin nodules nodule- confirmed RAAS with suspicious axillary nodes	Bilateral extended mastectomies with LD flap reconstruction	Multiple liver metastases Palliative care Dead one year following RAAS diagnosis
2	Bilateral metachronous (right followed by left breast cancer 3 years later)	Metachronous right followed by left RAAS a year later	7	Erythematous right breast lesions confirmed RAAS. Skin ulceration over left breast, confirmed RAAS a year later	Right extended mastectomy and integra and skin graft reconstruction Left extended mastectomy and skin graft reconstruction. Long healing due to exposed ribs	Dead one year following RAAS diagnosis while under surveillance
3	Bilateral synchronous screen detected breast ca	Metachronous, Right followed by left RAAS one year later	6	Presented with right skin discolouration, proven right RAAS	Right extended mastectomy, wide clearance of chest wall and LD FLAP construction Left extended mastectomy + LD flap reconstruction	Dead 9 months post op
4	Metachronous primary, left and then right breast cancer 32 yrs later	Synchronous in 2018	4	Skin lesions on both breasts confirmed RAAS	Bilateral extended mastectomies with Integra and skin graft reconstruction	Alive 6 years post op

^*^Integra is a skin substitute used when skin cover is not possible and is often used in burns and scars. It is made of silicone and bovine collagen and is Thought to aid healing process

### Latency from radiotherapy administration to RAAS development

**Fig. 1:**
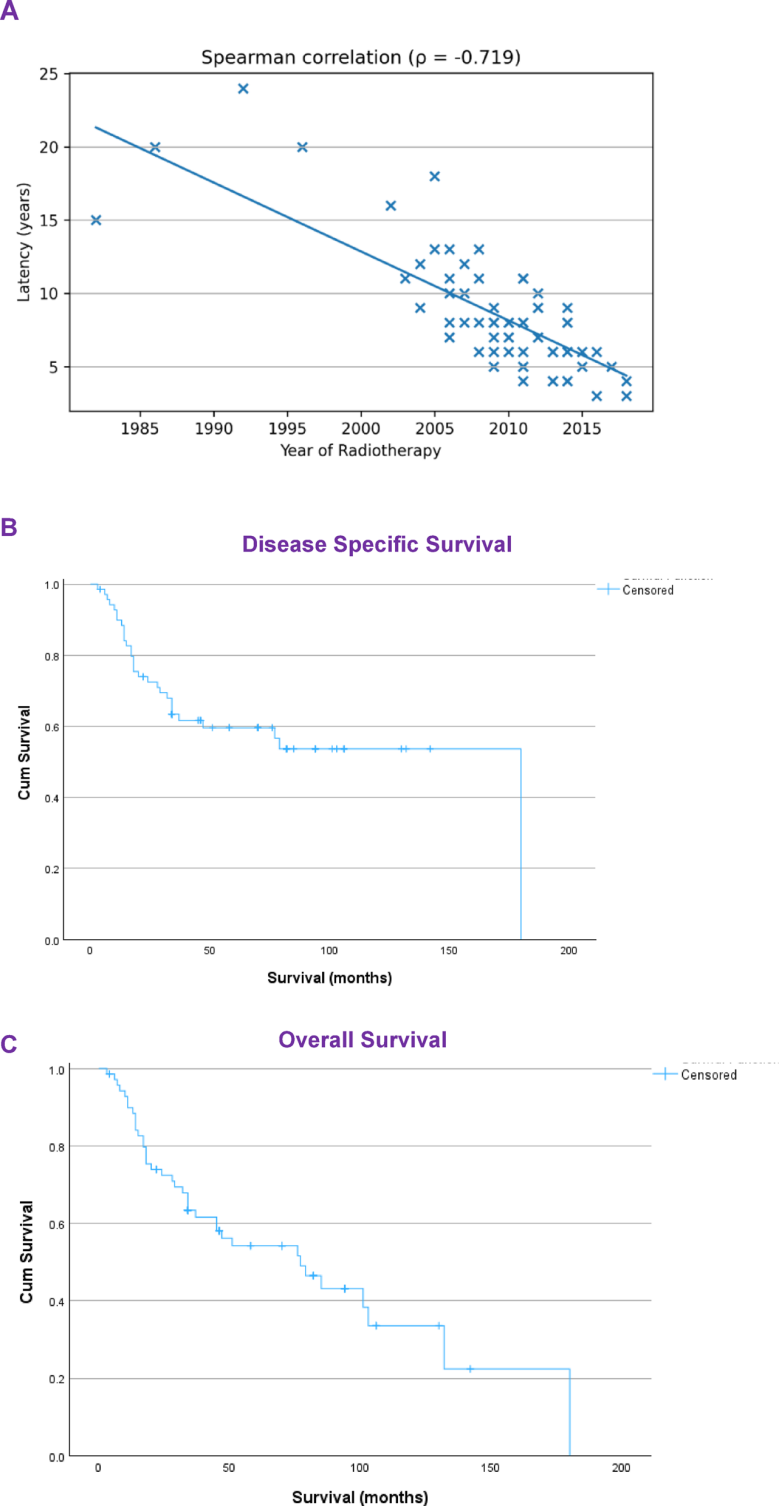
**A**. Latency between radiotherapy administration and diagnosis of breast RAAS over the years. **B**. Kaplan Meier plot for angiosarcoma specific survival. **C**. Kaplan Meier plot for angiosarcoma overall survival

RAAS cases included in the study were preceded by primary cancer diagnoses spanning from 1986–2018. The median latency from treatment of primary breast cancer to diagnosis of the first occurrence of RAAS was 8 years (range 3–24 years). The mean latency for bilateral RAAS patients was 6.5 years (range: 4–9 years). The relationship between the year of radiotherapy administration and the interval in which patients first developed angiosarcoma was investigated. The interval to RAAS diagnosis has significantly reduced over the years (r = − 0.719, *p* < 0.001), Fig. 1A.

### Treatment of RAAS angiosarcoma

The treatment options offered to the patients in this study were largely: surgery (extended mastectomy) alone, surgery and radiotherapy and surgery and palliative chemotherapy in cases with distance metastases (Table 1). Extended mastectomy involves removal of the breast (including nipple, overlying skin, radiation field and underlying pectoralis major muscle) with the aim of removal of as much as possible of the radiation field. In Bilateral angiosarcomas, double extended mastectomy was performed. Nodal sampling was not routinely performed unless there is suspicious imaging. In one case, neoadjuvant chemotherapy was administered due to the initial misdiagnosis as triple negative breast carcinoma at the local hospital. 90% of the patients underwent surgical resection; of whom only 4.2% received radiotherapy in addition.

### Recurrences and survival outcome of RAAS

Of the 70 patients, 22 patients (31%) had recurrences of angiosarcoma. Five patients (7%) had developed bilateral RAAS, of whom two patients had unilateral breast cancer preceding the RAAS and three patients had bilateral breast cancer. Only one of 4 patients with bilateral angiosarcomas remains alive (Table 2). Of the 22 patients who had recurrent angiosarcoma, 20 have subsequently died. On Kaplan-Meir analysis, the 5-year disease specific survival was 59.6%, and overall survival was 54.2%, Fig. 1B-C.

To date, 38 patients (54.3%) included in the study had died. The median age of death was 77 years old (range 58–96 years old). The median interval from the diagnosis of angiosarcoma to death was 26 months (range 3–180 months).

### The relationship between histological parameters and survival

Binary logistic regression showed increasing surgical resection margins microscopic width significantly reduced recurrence (OR = 0.935, *p* = 0.006) but cox regression analysis showed no significant direct impact on survival, HR = 1.002, *p* = 0.798. Recurrence was a strong predictor of mortality, HR = 2.856, *p* = 0.005. Resection margins of 10 mm or greater reduced recurrence risk significantly, *p* = 0.044, OR = 0.323. Where the information was available, the type of primary breast cancer, its grade or treatment had no bearing on RAAS latency or outcome.

Using multivariate Cox regression analysis, neither angiosarcomas’ grade nor other histological characteristics correlated with survival. No significant differences in the age at diagnosis or latency to developing RAAS were found between those who died and those who survived. No association was found between c-Myc positivity and survival rates.

### Primary angiosarcoma (PAS)

A total of nine patients with PAS were included in this study, with ages ranging from 18 to 69 years (median 31yrs). All cases presented as breast lumps; two of which were detected during routine screening. The Ki-67 proliferation index varied widely, with higher-grade tumors in PAS demonstrating indices of up to 90%.

Metastatic spread was observed in five (55.6%) patients at routine follow up, with common sites including the liver, bone, lung, and soft tissue, and four patients (44.4%) succumbed to the disease within two years of diagnosis. Lymph node involvement was documented in two patients, with one case showing three positive nodes and another a single metastatic node. At the time of write up, one patient is receiving additional chemotherapy for aggressive metastatic progression. Five patients had died, and three remain disease-free beyond two years postoperatively.

## Discussion

Angiosarcoma of the breast is a rare and aggressive disease with poor outcomes. There is limited existing literature on AS and minimal evidence base for the current recommended management. This retrospective study provides important insights into the presentation, management and prognostic factors for RAAS of the breast. Currently, the British Sarcoma Group (BSG) recommends all angiosarcoma patients to be discussed in a multidisciplinary team (MDT) meeting and surgical resection to be carried out by an appropriately trained surgeon who is a member of the sarcoma MDT [11].

RAAS is recognised as a late complication of adjuvant radiotherapy for breast cancer management. There has been an increase in the cases of RAAS reported in literature in recent years [18]. Our study found the median latency of RAAS development to be RAAS was 8 years (range 3–24 years), which is comparable to previous studies [16, 19–21]. A recent review of the literature reported that most cases developed 5–10 years following radiotherapy [18]. An intriguing finding in this cohort is that the latency of RAAS development appears to have shortened over the years. Similarly, in their study of 22 RAAS between 2010 and 2022 Wong et al. reported a decrease in the interval between breast radiotherapy and RAAS development over the last 24 years (r^2^ = 0.6601) [22]. Furthermore, in our study, this interval was shorter (6.5 years) for patients presenting with bilateral RAAS compared with the total population (8 years). Most of the latter had metachronous breast cancer, with each episode treated with adjuvant radiotherapy which may have increased the radiation dose received leading to acceleration of RAAS development. This finding, however, should be interpreted with caution as it may represent a selection bias in the retrospective design of this study. It is also possible that the perceived reduced latency reflects increased recognition of the disease and early diagnosis particularly in specialist units where rigorous pathology review is undertaken. Moe et al. reported a case with short latency of 3 years where the diagnosis was not initially made on the biopsy leading to delayed diagnosis and perceived longer latency [23]

There has been a number of changes to the dose, frequency, duration and field of adjuvant radiotherapy over the years including accelerated partial breast radiation [24–27].

The current recommended post-surgery radiotherapy regime is administration of 40 Gy in 15 fractions over 3 weeks for women with invasive breast cancer after breast‑conserving surgery or mastectomy. The increase in energy of radiotherapy linear accelerators may also be a contributor [21]. During the COVID-19 pandemic, the Royal College of Radiologists recommended an amended radiotherapy regime, considered to be equally effective with fewer fractions in order to minimise hospital attendance [28]. Overall, there has been a paradigm shift in managing early breast cancer towards de-escalation of radiotherapy. Partial breast irradiation (focusing on lumpectomy, cavity and adjacent tissue) has largely replaced total breast irradiation. Conventionally fractionated radiation (25–28 fractions) could now be substituted with hypofractionated (15–16 fractions) and ultra-hypo fractionated radiation (5 fractions) with a total lower dose (50 Gy vs 39–43 Gy vs 26–30 Gy) [29]. Those techniques, together with future developments such as precision cancer care and toxicity prevention, may modify the frequency and interval of RAAS development.

RAAS is known to have high recurrence rates and smaller resection margins are known to increase recurrence rate [20, 30]. Indeed, 28% of the patients included in this study had at least one recurrence. Gutkin et al. [31] recommended margins of greater than 5 mm for prevention of local recurrence. Salminen et al. [30] also observed a greater number of local recurrences with a surgical margin clearance of less than 10 mm. Currently, the consensus for adequate resection margin is at least 10 mm. Our study concurs that microscopic margins of less than 10 mm lead to significantly higher rates of recurrence (*p* = 0.001), highlighting the importance of adequate surgical clearance.

The main management option for RAAS is aggressive surgical resection [32]. At our institution, only primary angiosarcomas are offered adjuvant radiotherapy for local control. The poorer outcome of patients with larger tumours may reflect a more aggressive biology and suggests the need for adjuvant chemo or radiotherapy in these patients. Adjuvant radiotherapy has not been shown to improve survival in existing literature [21, 33], however, a systematic review has shown adjuvant radiotherapy is beneficial in preventing local recurrence in RAAS [34]. Newer research on radical resection of the entire irradiated field in RAAS has shown to improve local control rates [35] since it could be argued that despite aggressive surgery, recurrences may still occur due to the field effect of radiotherapy and failure to excise the whole radiation field [16]. This may also explain the lack of association between margin width and survival. Aggressive surgical management of RAAS with removal of the irradiated skin and muscle has been proposed in earlier small series reports [36, 37]. Our institution performs extended mastectomies with the aim of removing as much of the irradiated field as possible. All patients in the current study, including those with no documented muscle involvement, underwent extended mastectomy and excision of the pectoralis major muscle. The aim of surgery was to resect as much of the radiotherapy field as possible and including the pectoralis major muscle. We previously provided evidence for this approach by comparing outcomes of radical excision of the irradiated field in our specialist sarcoma service to those managed at local hospitals with less extensive surgery [12]. Specialist management of RAAS led to fewer local recurrences (9 of 26 vs 8 of 10; *p* = 0.015) and improved disease-specific survival; 91.1 (range 69.2–113.0) vs 48.8 (18.6–79.1) months respectively (*p* = 0.012) [12]. Similarly, In their study of 49 women, including 26 managed by a sarcoma specialist team and 23 by local teams, Guram et al., showed that management by a sarcoma multidisciplinary team was associated with more extensive surgery (*p* = 0.0000) and better 3-year recurrence free survival (*p* = 0.019) [38]. A recent Australian sarcoma unit experience and metaanalysis provided further evidence supporting this approach [32]. A total of 22 patients who underwent extended resection of RAAS, with a median follow-up of 33.5 months, had 3-and 5-year DFS rate of 75.2% for both. On comparing their figures with systematic review data from 17 eligible studies of less aggressive surgery, the authors confirmed that the extended approach was associated with a low rate of local recurrences [32].

While it is not currently our standard practice, neoadjuvant chemotherapy (NACT) has emerged as a potential therapeutic option for mammary angiosarcoma to improve rates of R0 margin with promising results. A recent single center Dutch study of 35 RAAS patients, 37% of whom received NACT, reported pathological complete response in 9 patients and improved distant metastasis free survival and OS in patients who underwent NACT [39].In their study of 29 patients (4 PAS, 24 RAAS), Chang et al. reported better DFS for good responders (*p* = 0.04). The OS survival in the NACT group correlated with tumour size (> 10 cm) in multivariate analysis [40].

Taxane based NACT has been standard for cutaneous angiosarcoma with Paclitaxel the most widely used chemotherapeutic agent [41]. The landmark trial ANGIOTAX of metastatic or unresectable cutaneous angiosarcoma showed objective response rate and disease control rate of 19% and 74% respectively [42]. A review of literature demonstrated a role for NACT in downsizing and achieving clear margins in primary cutaneous and cardiac angiosarcomas although no impact on survival was identified [43]. A large multi-institutional series of 86 patients soft tissue angiosarcomas, includes 43 treated with NACT and 16 treated with NACT and adjuvant chemotherapy, reported outcomes comparable with the published data and proposed a randomized trial to provide firm evidence in this area [44]. A trimodality approach of taxane induction, concurrent taxane/radiation followed by surgical resection with wide margins of breast RAAS provided superior local control, higher pCR rates and better 5-year recurrence free survival RFS compared with monotherapy; 93.8% vs. 42.9%; *P* = 0.004 [45]. It is of note that one of our patients who presented with bilateral metachronous angiosarcoma was treated weekly Paclitaxel chemotherapy for the recurrent tumour and remains alive and well with no evidence of recurrence 9 years following the diagnosis.

With regards to patient survival, reported figures have been highly variable, with the overall 5 year survival rates ranging from 10 to 75% [46, 47]. Some studies have found parameters such as history of recurrence and tumour grade to affect survival [20, 33]. The disparity in survival rates is attributed to the heterogeneity of the RAAS and the lack of its standardised management. The most recent UK national study (Breast Angiosarcoma Surveillance Study; BRASS) [16] reported a 45% estimated 5-year survival rate. A previous report from our institution [12] demonstrated superior patient outcomes of patients managed at a tertiary Sarcoma centre as opposed to local hospitals. This current study of patients managed at our Breast and Sarcoma centre show the patients’ overall 5 year survival to be approximately 60% which is superior to the UK national figures [16], Dutch data [21] and well above the collective figure of 32% reported in a systematic review by Depla et al. [34]. These figures support the BSG’s recommendation for all RAAS patients to be discussed at a specialist MDT and suggest that in addition to discussion at MDT, patients would benefit from accurate diagnosis, specialist discussion and management given the improvement demonstrated in patient outcomes.

We report a much younger age at presentation for PAS compared with RAAS (median 31 vs 71 respectively). PAS has consistently been reported to occur in younger patients [31, 48]. Previous studies have suggested c-Myc to be a useful marker for distinguishing between benign vascular lesions and AS, and also between PAS and RAAS using fluorescent in situ hybridization (FISH) [48, 49]. Some studies have reported that c-Myc expression was associated with shorter survival and poorer outcomes in angiosarcomas [50, 51]. Our current study did not show significant correlation between c-Myc immunohistochemical expression and survival.

## Conclusion

In summary, this is a large series of an uncommon malignancy managed at a dedicated sarcoma tertiary referral center. It further characterises the presentation and behaviour of both PAS and RAAS and provides insight into potential clinically relevant factors that affect patient outcome. Aggressive surgery (extended mastectomy with removal of underlying pectoralis major and the skin in the radiation field) is recommended. Close resection margins correlated with disease recurrence and mortality, respectively. Our data supports a microscopic margin of at least 10 mm. Local recurrences were associated with high likelihood of mortality from the disease. Age, tumour grade, tumour size and c-Myc expression did not correlate with survival in RAAS.

## Data Availability

Dataset not publicly available due to patient confidentiality but anonymised data will be made available upon reasonable request to the corresponding author.
